# Improved Performance, Seed Germination and Degradation Behavior of Bamboo Fiber Paper Mulch Film Reinforced by Nano Bacterial Cellulose

**DOI:** 10.3390/polym18070815

**Published:** 2026-03-27

**Authors:** Xu Liu, Ying Li, Siyu Liu, Mingjie Guan, Shuai Qian, Fei Xiao, Cheng Yong, Mengyu Wu, Pulin Che

**Affiliations:** 1 College of Materials Science and Engineering, Nanjing Forestry University, Nanjing 210037, China; 2 Bamboo Research Institute, College of Forestry and Grassland & College of Soil and Water Conservation, Nanjing Forestry University, Nanjing 210037, China; 3 College of International Education, Nanjing Forestry University, Nanjing 210037, China; 4 Hunan Academy of Forestry, Changsha 410004, China; 5 Institute of Agricultural Resources and Environment, Jiangsu Academy of Agricultural Sciences, Nanjing 210014, China

**Keywords:** bamboo residue, fiber paper mulch, seed germination, degradation behavior, bacterial cellulose

## Abstract

To address the limitation of insufficient mechanical strength and short service life in biodegradable bamboo fiber mulch film (BFM) replacing plastic film in agriculture, this study applied a biochemical method to make bamboo fiber and used bacterial cellulose (BC) as a natural nanoscale reinforcing agent to fabricate high-performance bacterial cellulose bamboo fiber mulch film (BC-BFM). The physical and mechanical properties, chemical structure, seed germination and degradation behavior performance of BC-BFM were characterized. Results demonstrated the structural compactness and homogeneity of the BC-BFM were improved markedly with the increase in BC addition and BC formed a 3D nanofibrillar network that effectively bridged inter-fiber voids. The tensile, burst and tear indexes of BC-BFM all significantly rose with BC addition. Notably, compared to plastic film and BFM, BC-BFM exhibited a good effect on mung bean seed germination and the best growth speed was at 5% BC addition. Furthermore, the degradation test showed that the degradation rate of BC-BFM within 90 d was three times less than that of BFM and service life was similar to plastic film. This showed that it was a promising method to prepare biodegradable high-quality BFM through biochemical preparation of bamboo fiber and BC nanocellulose reinforcement. This method markedly enhanced the mechanical performance and durability of BC-BFM, providing a feasible technical path for the development of biodegradable high-performance green agricultural covering materials with long service life.

## 1. Introduction

The non-biodegradability of conventional plastic mulches such as polyethylene (PE) and polyvinyl chloride (PVC) has given rise to a severe environmental issue known as “white pollution” in China [1,2,3], whereby the annual residual accumulation of agricultural plastic film reaches approximately one million tons [4,5]. Currently, fiber-based paper mulch has emerged as a focus in biodegradable agricultural mulching materials [6,7]. With favorable mechanical properties and functional performance, fiber-based paper mulch offers a viable technical pathway for the advancement of high-performance, biodegradable, and environmentally friendly agricultural covering materials [8].

Bamboo is one of the fastest-growing plants in the world, whose maximum daily growth height can exceed 1 m [9]; it represents a widely available and extensively utilized biomass resource [10]. During the processing and manufacturing of bamboo-based materials, over 46 million tons of bamboo residue is generated annually [11,12]. This byproduct possesses favorable physical properties such as high strength-to-weight ratio and is composed primarily of cellulose, hemicellulose, and lignin, exhibiting inherent biodegradability [13]. Despite these advantageous characteristics, bamboo residue remains largely underutilized and inadequately recycled, with the majority currently disposed of through environmentally harmful practices such as open incineration [14]. This not only resulted in the loss of valuable biomass resources [15] but also contributed to environmental pollution [16]. In light of the growing advocacy for the “bamboo as a substitute for plastic” initiative, bamboo fiber-based paper mulch film has emerged as a promising candidate for replacing conventional non-biodegradable plastic mulching materials.

However, the fiber paper mulch films exhibit lower tensile, burst strength than conventional plastic mulch films. Therefore, these films are prone to mechanical damage and environmental stress breakage resulting in a short service life during application. To improve their mechanical performance, reinforcing agents like urea-formaldehyde resins (UF), melamine-formaldehyde resin (MF), and Polyamide epichlorohydrin (PAE) are commonly added to the fiber pulp [17,18,19]. However, these synthetic additives have limited biodegradability and may release harmful byproducts upon degradation. In contrast, environmentally friendly biodegradable additives, such as starch [20], chitosan [21], cellulose derivatives [22], and plant gums [23], have attracted extensive attention.

BC is a naturally nanostructured, biodegradable polymer produced by acetic acid bacteria, free from lignin, pectin, hemicellulose, and other impurities found in plant cellulose, resulting in higher chemical purity [24,25,26]. BC exhibited an ultrafine fibrillar network, high crystallinity, excellent water retention, and exceptional mechanical strength [27,28,29]. Its molecular structure consists of cellulose ([-C_6_H_10_O_5_-]_n_), which can be efficiently broken down into glucose by cellulase enzymes under natural conditions, ensuring complete biodegradability [30,31]. Xiang [32] demonstrated that paper produced from recycled fibers exhibited a 12.7% increase in dry tensile strength when utilizing dispersed BC fibers. Campano [33] incorporated low-fibrillated bacterial cellulose nanofibers (BCNF) into recycled pulp composed of a mixture of old newsprint (60%) and old magazine paper (40%); the results indicated improvements in all measured mechanical properties: the tensile index increased by 11.1%, the tear index by 7.6%, and porosity was reduced. While previous studies have explored BC as a reinforcement agent, a systematic investigation into the quantitative structure–property relationship across a wide range of BC additions (1–9%) in bamboo fiber mulch films has not been reported.

Therefore, the present study aims to address the urgent need for sustainable alternatives to synthetic plastic mulches by developing a novel biodegradable mulch film based on bamboo fibers reinforced with BC. This study prepared bamboo fibers using a bio-fermentation/low-alkali pretreatment and added varying BC additions to fabricate BC-BFM. The addition effect of BC on the performance of BC-BFM was systematically studied. The functional group variations, crystallinity evolution and the microstructural morphology of BC-BFM were characterized. The drainability of fiber pulp; physical mechanical properties including tensile, tear and burst indexes; and physical properties such as air permeability, contact angle, surface color, and whiteness were comprehensively evaluated. Additionally, seed germination trials were conducted to assess agronomic applicability. Degradation experiments were further performed to investigate the degradation rate of BC-BFM under simulated raining season. This approach enables a thorough understanding of the relationship between BC addition and the overall performance and environmental degradability of BC-BFM, thereby providing theoretical guidance for the development of advanced biodegradable mulching films based on BC-enhanced bamboo fibers and offering a viable and eco-friendly alternative to conventional plastic mulches.

## 2. Materials and Methods

### 2.1. Materials

Bamboo residues from the floor processing of 4–6-year-old Moso bamboo were used as the raw fiber material. The residual material was naturally air-dried under well-ventilated, dry, and controllable environmental conditions (20 °C, RH 65%) until the moisture content stabilized within the range of 8 ± 1%. To prepare the BC-BFM, the material with a sieve particle size of 10-mesh specification was selected as the raw fiber material.

Sodium hydroxide and urea were from Shanghai Al-addin Bio-chemical Tech. Co., Ltd., Shanghai, China.

The commercial PE plastic mulch was purchased from market with a thickness of 0.01 mm.

**Figure 1:**
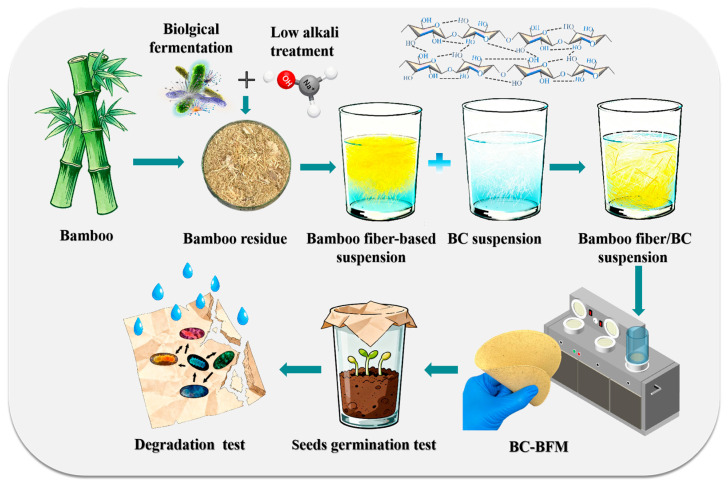
Schematic demonstrating bamboo fiber biological deconstruction, in preparation for BC suspension fabrication of BC-BFM seed germination test and degradation test.

The microbial strains involved in the research (*Aspergillus oryzae*, *Bacillus subtilis*, *Streptomyces rochei* and *Bacillus licheniformis*) were provided by Ning-liang Bio-Engineering Co., Ltd., Nanjing, China. Figure 1 shows the overall technical route of this work.

### 2.2. Bamboo Residue Biochemical Pretreatment

#### 2.2.1. Biological Pretreatment

First, 2000 g of bamboo waste powder measured on an absolutely dry weight basis was loaded into the aerobic composting reactor (ZKNK20031, Jiangsu Zhongke Nuke Ecological Technology Co., Ltd., Nanjing, China). Distilled water was intermittently sprayed onto bamboo residue to make the moisture content of the substrate 55 ± 3%. The substrate and a microbial inoculant at a dosage equivalent to 3% wt of the absolute dried bamboo residue were mixed together under the carbon-to-nitrogen C:N ratio of 30:1 by supplementing with urea, and the mixture were fermented at a temperature of 60 °C throughout the process. Daily maintenance included adding water to maintain moisture levels and stirring the substrate evenly. After 12 d, the fermented bamboo residue was collected for the next treatment [34].

#### 2.2.2. Low-Alkali Treatment

The fermented bamboo residue was suspended in water to achieve a material-to-water rate of 1:10. It was then treated with 1% sodium hydroxide under high-temperature and mild-alkaline conditions using steam cooking. The process was carried out in a portable pressure steam sterilizer (XFS-280A, Shanghai Shangpu Instrument and Equipment Co., Ltd., Shanghai, China) at 120 °C for 1 h; then, the pH of treated bamboo reside was adjusted to near 7.0, thereby yielding the pretreated bamboo fibers with chemical content as follows: cellulose content of 59.13%, hemicellulose content of 15.14%, lignin content of 21.00% and ash content of 1.05%.

### 2.3. Preparation of BC Suspension

Next, 35 ± 5 g of wet BC tablets was cut into pieces measuring 20 mm × 20 mm × 3 mm. The BC pieces were subsequently immersed in 10 times their volume of deionized water and homogenized using a laboratory tissue homogenizer (JJ-2B, Suzhou Surui Instrument Co., Ltd., Suzhou, China) at a speed of 5000 rpm for 4 min. The resulting suspension was then transferred to a beaker and further homogenized using a digital high-speed disperser (FJ200-SH, Shanghai Bosheng Model Factory, Shanghai, China) at 15,000 rpm for 3 min to obtain a BC suspension. After dilution, the concentration of the BC suspension was 0.428%. The surface morphology of wet BC tablets and BC suspension was observed using a scanning electron microscope (SEM, Quanta-200 model from Tokyo, Japan), with a scanning voltage of 15 kv.

### 2.4. Preparation of BC-BFM and Drainability of Fiber Pulps

The pretreated bamboo fiber was refined using a disk refiner with a gap of 0.2–0.5 mm and this refining process was repeated for 3 cycles to obtain bamboo fiber pulp. The bamboo fiber pulp was washed, deaerated, and concentrated. The base weight of the bamboo fiber pulp handsheet was 60 g/m^2^. BC suspension was mixed into the bamboo fiber pulp at varying additions (0%, 1%, 3%, 5%, 7%, and 9%) based on the absolute drying weight of bamboo fiber. Therefore, the dry weights of BC added to each bamboo fiber-based suspension were 0.012 g, 0.036 g, 0.060 g, 0.084 g, and 0.108 g, respectively. The sample with 0% of BC addition was designated as BFM, and the BC additions 1% to 9% BC were labeled BC-BFM.

In accordance with GB/T18402-2001 [35] “Pulps—Determination of drainability (drainage time method)”, the drainage performance of the mixed bamboo fiber pulp water suspension at 20 °C was measured by means of a standard paper sheet former with a diameter of 200 mm. The unit of measurement was expressed in seconds (s). The test was carried out 5 times, and the average value of drainage time was calculated.

Handsheets were prepared according to TAPPI T205 sp-06 standard use standard paper sheet former at a temperature of 110 ± 2 °C under a pressure of 2.0 MPa for 10 min and hot-pressed into bamboo fiber mulch with a thickness of 150 ± 0.75 μm. All samples are conditioned for 24 h under standard atmospheric conditions (23 °C, 50% relative humidity) prior to physical strength testing [36].

### 2.5. Properties of BC-BFM Test

#### 2.5.1. Tensile Strength

The tensile strength of the BFM and five groups of BC-BFM was assessed following the “Paper and Board—Determination of Tensile Properties—Constant Rate of Elongation Method (20 mm/min)” protocol outlined in the standard ISO 1924–2:2010 [37]. Testing was carried out by a tensile tester (Z B–WLQ 300, Zhi Bang Auto. Tech. Co., Ltd., Hangzhou, China) at a loading speed of 20 mm/min. Each specimen measured 10 mm (width) × 150 mm (length), and 5 replicates were tested for statistical reliability. The results were expressed as the tensile index (N·m/g), which was calculated according to Equation (1).

(1) $$tensileindex=\frac{S}{g}\times 10^{3}$$

where g is the paper grammage (g/m^2^) and S is the tensile strength (kN/m).

#### 2.5.2. Tearing Resistance

Tear resistance tests were performed on BFM and five groups of BC-BFM in compliance with the standard ISO 1974:2012 [38], titled “Paper and Board—Determination of Tearing Resistance.” A microcomputer-controlled tester of tearing resistance (Model AT–SL-2, ANnimet Instrument Co., Ltd., Shandong, Jinan, China) was employed for the measurements. Each sample had dimensions of 63 mm length and 50 mm width, and 5 replicates were tested to ensure reliability. The tearing resistance was expressed as the tear index (mN·m^2^/g), calculated using the formula provided in Equation (2).

(2) $$tear\;index=\frac{F}{g}$$

where g is the paper grammage (g/m^2^) and F is the tearing resistance (mN).

#### 2.5.3. Bursting Strength

The bursting strength of the BFM and five groups of BC-BFM was assessed using a HH-NP1600A tester of bursting strength (HuaHan PaperMaking Testing Instrument & Equipment Co., Ltd., Hangzhou, China), following the ISO 2758:2014 [39] titled “Paper—Determination of Bursting Strength.” Each specimen was prepared with a diameter of 200 mm, and 5 replicates were conducted for each sample. The burst strength was quantified via the burst index (kPa·m^2^/g), with its calculation formula detailed in Equation (3).

(3) $$burst\;index=\frac{p}{g}$$

where g is the paper grammage (g/m^2^) andp is the average bursting strength (kPa).

#### 2.5.4. Measurement of Surface Contact Angle

The contact angle tester (JC2000D5, Shanghai Zhongchen Digital Technology Equipment Co., Ltd., Shanghai, China) was employed to measure the static contact angles of distilled water (a polar liquid) on the surfaces of the BFM and five groups of BC-BFM. Measurements were conducted at 5 randomly selected points for each sample, with a liquid droplet volume of 5 μL, and the contact angle was recorded from the first image captured immediately upon droplet contact with the surface (approximately 1 s after deposition). The same procedure was applied to determine the static contact angles of dimethyl methane (a non-polar liquid) on the surface of each of the BFM and five BC-BFM. Surface energy was subsequently calculated using the Owens two-liquid method.

#### 2.5.5. Surface Color and ISO Brightness Measurement

The colorimetric measurements were conducted using a colorimeter (ZB-A, Hangzhou Paperbang Automation Technology Co., Ltd., Hangzhou, China) to evaluate the L*, a*, b* and ISO brightness values of the BFM group and five groups of BC-BFM. Measurements were taken at 5 randomly selected points for each sample. The L* parameter represents lightness, changing from black (0) to white (100), where higher number shows greater brightness [40]. The a* value, ranging from −128 (green) to +127 (red), indicated the chromaticity along the green–red axis; an increase in a* from negative to positive values corresponds to a shift from greenish to reddish hues. The b* value, ranging from −128 (blue) to +127 (yellow), reflects the chromaticity along the blue–yellow axis; an increase in b* from negative to positive values indicated a transition from bluish to yellowish tones.

#### 2.5.6. Air Permeance of BC-BFM Test

In accordance with standard ISO 5636-1:1984 [41] “Paper and Board—Determination of Air Permeance”, the air permeability was tested using a BendtSen Air Permeability Tester under a standard pressure of 1.47 kPa. The BFM and five groups of BC-BFM were firmly clamped between the annular plate and the sealing gasket, and the flowmeter reading was recorded 5 s after clamping. Based on Equation (4), the Bendtsen air permeability (P) was calculated, with its unit expressed as (μm/Pa·s). The average value was calculated from 5 samples of each group.

(4) P=0.113q

where P is the air permeability at a standard pressure difference of 1.47 kPa (μm/Pa·s) and q is the air volume passing through the test surface of the sample per minute (mL/min).

### 2.6. Characterization of BC-BFM

#### 2.6.1. FTIR Spectra and XRD Test

The BFM and five groups of BC-BFM were dried in an oven for 24 h at 60 °C. Vertex 80 V spectrometer of FTIR (BRUKER, Mannheim, Germany) was employed to characterized potentially chemical bonds changes in the BFM and five groups of BC-BFM. Spectra were recorded over 4000–400 cm^−1^ at 0.06 cm^−1^ resolution.

Then, 20 mm × 20 mm samples of the BFM and five groups of BC-BFM were dried for 24 h at 60 °C. The crystallinity of the fibers was analyzed by XRD using a D8 Advance—Diffracto-meter (BRUKER, Mannheim, Germany) at 40 mA and 40 kV. Diffraction spectra were captured over a 2θ angular range of 5° to 40° with a scanning speed of 10°/min. The crystallinity index (CrI, %) was determined using Equation (5) [42].

(5) $$CrI=\frac{I_{002}-I_{am}}{I_{002}}\times 100\%$$

where $I_{002}$ is the peak intensity at 2θ = 22° and ${\;I}_{am}$ is the background scattering intensity at 2θ = 18.0°.

#### 2.6.2. Surface/Cross-Section Micromorphology

Samples measuring approximately 8 mm × 8 mm were obtained from the BFM and five groups of BC-BFM. The cross-section microstructure and surface morphology of these specimens were observed by a scanning electron microscope (SEM) (Quanta-200, Japan) at an accelerating voltage of 15 kV.

### 2.7. Seed Germination

Following Liang’s report [43], the air permeability and application potential of BFM and five groups of BC-BFM were evaluated via soybean germination assays using healthy seeds pre-soaked for 24 h at 28 °C; garden soil was transferred into transparent plastic cups. Five soybean seeds were sown per experiment and a seven-group treatment was designed: the black plastic film (plastic), the BFM and five BC-BFM groups. Soybean sprout emergence was monitored daily, and sprout stem length was measured on the 4 d after sowing.

### 2.8. Degradation Behavior of BC-BFM

The 100 mm diameter black plastic film, BFM and five groups of BC-BFM were placed on the soil surface within Petri dishes; the soil was collected from the garden soil within the Jiangsu Academy of Agricultural Sciences in Xuanwu District, Nanjing City, Jiangsu Province. The soil type was sandy loam. It was collected from the top 20 cm of the soil surface. The pH value was 6.2 ± 0.3 and the moisture content was 45% ± 3%; the relative humidity was maintained at 75–85% and the temperature was kept at 20 ± 5 °C. Morphological images of the BC-BFM surface were captured at 15–30 d intervals. After 90 d, the surface degradation rate of BC-BFM in each photo was analyzed using the ImageJ 1.8.0 software. At the same time, the BC-BFM samples after degradation for 90 d were taken out, washed, dried to a constant weight, and weighed to calculate the weight loss rate (WLR, %) using Equation (6). Subsequently, the morphology of BC-BFM after degradation was observed using a scanning electron microscope (with the model of Japanese Quanta-200).

(6) $$WLR=\frac{w_{0}-w_{1}}{w_{0}}\times 100\%$$

where $w_{0}$ is the mass of the sample before degradation and ${\;w}_{1}$ is the mass of the sample after degradation for 90 d.

## 3. Results

### 3.1. The Structure and Morphology of BC

**Figure 2:**
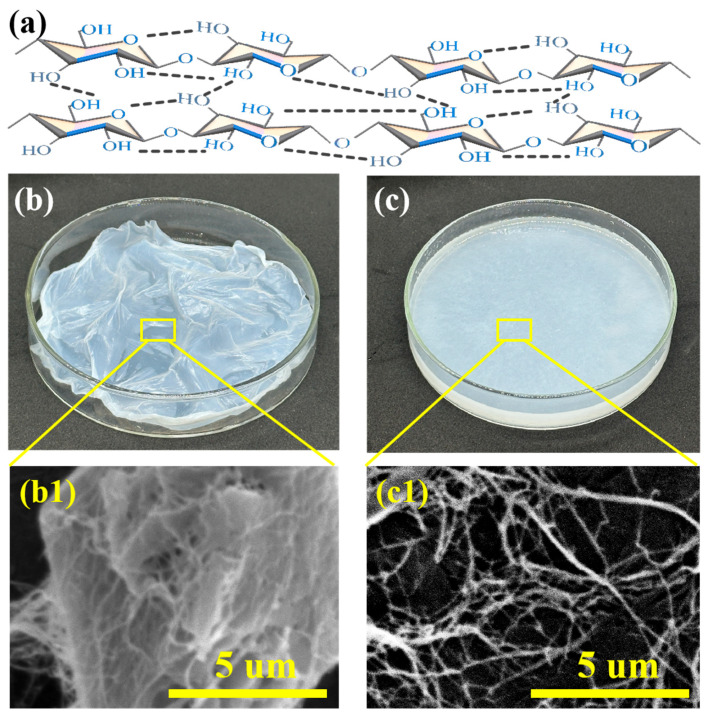
(**a**) The structural formula of BC. (**b**) The wet BC tablets before dispersion treatment. (**c**) The dispersed BC suspension. (**b1**) The SEM of wet BC tablets before dispersion treatment. (**c1**) The SEM of dispersed BC suspension.

Figure 2a shows the chemical structure of BC, which featured intramolecular and intermolecular hydrogen bonds within the molecule. As can be seen in the figure, the surface of BC contains abundant hydroxyl groups, endowing it with strong hydrophilicity. These hydroxyl groups provide very favorable conditions for the modification of BC. Figure 2b shows the morphology of wet BC tablets. As shown in Figure 2(b1), the BC fibers were agglomerated in a block-like manner. Figure 2c depicts the suspension state of BC fiber after mechanical dispersion of the wet BC film. Figure 2(c1) illustrates that the mechanically dispersed BC fibers were intertwined, forming a nano-spatial network structure. The diameter of the BC fiber ranged from 10 to 100 nm, and its length was greater than 10 μm.

### 3.2. Mechanical Property Analysis

**Figure 3:**
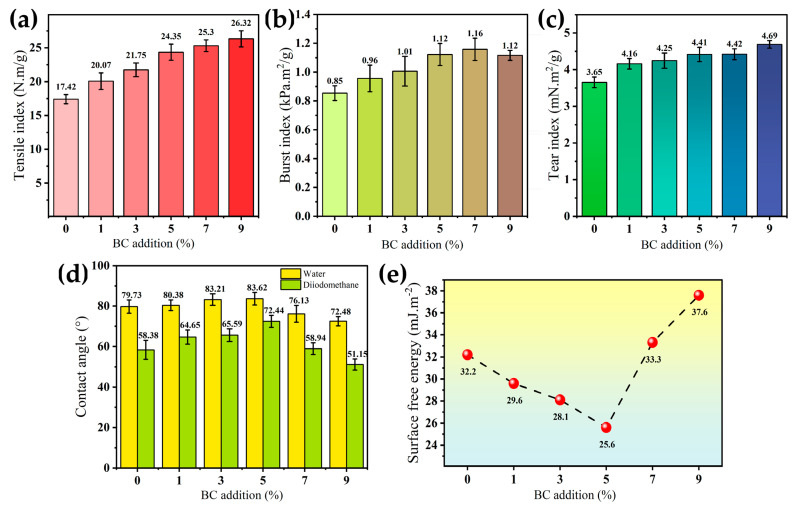
(**a**) Tensile index. (**b**) Burst index. (**c**) Tear index of BFM and five groups of BC-BFMs. (**d**) Surface contact angle of BFM and five groups of BC-BFMs. (**e**) Surface free energy of BFM and five groups of BC-BFMs.

As illustrated in Figure 3a–c respectively, all mechanical properties including tensile index, burst index, and tear index exhibited significant improvement following the addition of BC. Specifically, Figure 3a demonstrates that the tensile index increases steadily from 17.42 N·m/g to 26.33 N·m/g, representing an approximate enhancement of 51.5%. These effects should be attributed to interweaving function and hydrogen bonding between BC nanofiber and bamboo fermented fiber. As confirmed in Figure 2(c1), BC consists of a large number of nanofibers with a high aspect ratio (diameter ranging from 10 to 100 nanometers, and length greater than 10 μm), enabling BC nanofiber woven microscale fermented bamboo fiber to form the dense fiber film and achieve effective stress transfer between nanofiber and microfiber in BC-BFM. Meanwhile, the interfacial bonding among the microscale bamboo fibers was enhanced by the abundant hydrogen bonds provided by the BC nanofibers. Consequently, the addition of BC significantly improved the strength of the BC-BFM composite.

In Figure 3b, a slight reduction in burst strength was observed at a 9% addition level, potentially due to excessive BC addition causing over-compaction and non-uniform distribution within the pulp, leading to minor deterioration in burst performance [44]. Figure 3c indicates that the tear index showed a positive correlation with the BC addition; the robust hydrogen bond network formed by BC significantly strengthens inter-fiber bonding, increasing resistance to bamboo fiber pull-out. However, when the BC addition reached 7–9%, the growth rate of tear index lightly slowed down, which may be attributed to the limiting effect of the BC addition.

### 3.3. Surface Contact Angle and Surface Free Energy

As shown in Figure 3d,e, with the increasing of BC addition from 0% to 5%, the water contact angle of BC-BFM exhibited an upward shift, rising from 79.73° to 83.62°, while the surface free energy decreased from 32.2 mJ·m^−2^ to 25.6 mJ·m^−2^, indicating enhanced hydrophobicity of the BC-BFM surface. Although both BC and bamboo fibers contain hydrophilic functional groups (e.g., hydroxyl groups), at low addition levels, BC should mainly act as a filler that forms a compact interface with bamboo fibers, effectively filling microscale pores between bamboo fibers, thereby resulting in a smoother surface morphology. For hydrophilic surfaces (with contact angles less than 90°), a reduction in surface roughness typically led to an increase in water contact angle—indicating increased hydrophobicity—and simultaneously reduced the exposure of hydrophilic groups, consequently lowering surface free energy. However, when the BC addition exceeded 5%, the water contact angle of BC-BFM markedly decreased to 76.13° (7% of BC) and 72.48° (9% of BC), and the surface free energy increased to 33.3 mJ·m^−2^ and 37.6 mJ·m^−2^, respectively, reflecting a resurgence in hydrophilicity. This result may be related to the surface morphology: When the surface at a 5% BC content became more and more uniform, resulting in a higher contact angle. Meanwhile, at 7% and 9% contents, signs of BC aggregation appeared on the surface of BC-BFM, which increased the surface roughness and led to a decrease in the contact angle.

### 3.4. Surface Color and ISO Brightness Difference

**Figure 4:**
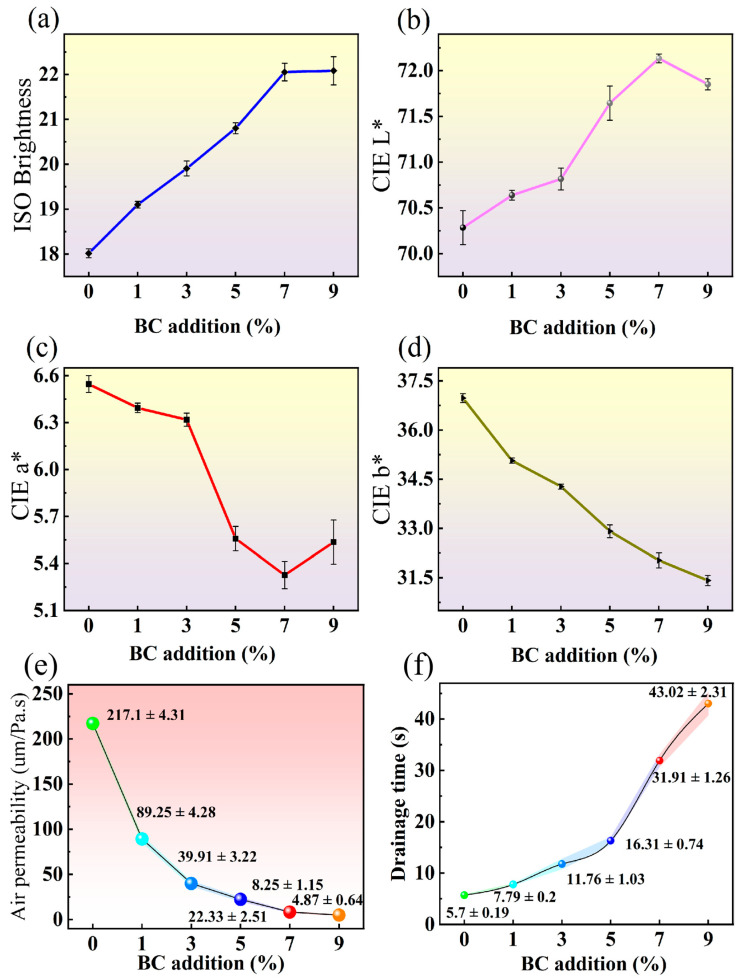
(**a**) ISO brightness; (**b**) CIE L*; (**c**) CIE a*; (**d**) CIE b* of BFM and five groups of BC-BFMs; (**e**) BFM and BC-BFM’s air permeability; (**f**) Drainage time of bamboo fiber pulp.

As illustrated in Figure 4a–d, the addition of BC led to a brighter, lighter and whiter BC-BFM appearance. Specifically, as illustrated in Figure 4a, the ISO brightness of BC-BFM increased from 18.02 (0% of BC) to 22.08 (9% of BC). ISO brightness was defined as the measurement of directional reflectance of a sample under blue light at a specific wavelength (457 nm) [45,46]. This parameter is particularly sensitive to yellow discoloration, as higher yellowness resulted in greater absorption of blue light and consequently lower ISO brightness values. Therefore, the addition of BC enhanced the visual quality of the BC-BFM without the need for chemical bleaching agents [29].

As shown in Figure 4b, the L* value of BC-BFM increased from 70.28 (0% of BC) to 72.13 (7% of BC), followed by a slight decrease at 9% of BC. This increase in luminance reflected enhanced surface reflectivity, which could be attributed to the formation of a smoother and more optically uniform surface upon BC addition. The minor reduction in L* at 9% may result from excessive BC addition, leading to uneven dispersion and slight agglomeration within the bamboo fiber pulp matrix.

Figure 4c showed that the a* value decreased from 6.55 (0% of BC) to 5.33 (7% of BC), with a slight rebound to 5.54 at 9% of BC. All measured a* values remain positive, indicating a progressive reduction in the redness of the film with increase in BC addition. As depicted in Figure 4d, the b* value decreases significantly from 36.98 (0% of BC) to 31.41 (9% of BC), demonstrating a pronounced reduction in yellowness. The initial yellowish hue of unbleached bamboo fibers is primarily due to chromophoric components such as lignin, which absorbs blue light. BC, characterized by high crystallinity, effectively counteracts the inherent yellow and red tones of bamboo fibers when incorporated into the matrix, resulting in the observed reductions in both a* and b* values.

### 3.5. Air Permeability and Drainage Time

Figure 4f illustrates the effects of BC addition on the drainage time of the bamboo fiber pulp and the air permeability of BC-BFM. The increase in BC addition exerted a progressively increasing effect on the drainage time of bamboo fiber pulp. The drainage time of BFM without BC was 5.7 s, which could be attributed to the relatively coarse bamboo fibers with micro-size interweaving the larger pore structure, facilitating rapid water drainage. BC possesses a high specific surface area and abundant hydrophilic hydroxyl groups on its surface, which formed hydrogen bonds with bamboo fiber, forming a micro-nano network and thereby obstructing water flow channels. During the initial dehydration phase, BC rapidly forms a dense “pre-coating” layer on the filter mesh, effectively prolonging the filtration time. When the BC addition exceeded 5%, the filtration time increased sharply, suggesting a substantial rise in energy requirements for dewatering [47].

Air permeability, defined as the ease with which air passes through the film, decreases significantly with BC addition. Figure 4e illustrates that when the BC was added into the BFM, bamboo fibers and nano BC fibers reconstructed a refined micro-nanoscale network that markedly reduced pore size and increased the tortuosity of gas diffusion pathways [48]. Consequently, the air permeability value declined from 217 μm/Pa·s for BFM (0% BC) to 4.9 μm/Pa·s—a reduction exceeding 90% [49].

### 3.6. FTIR and XRD Analysis of BC-BFM

**Figure 5:**
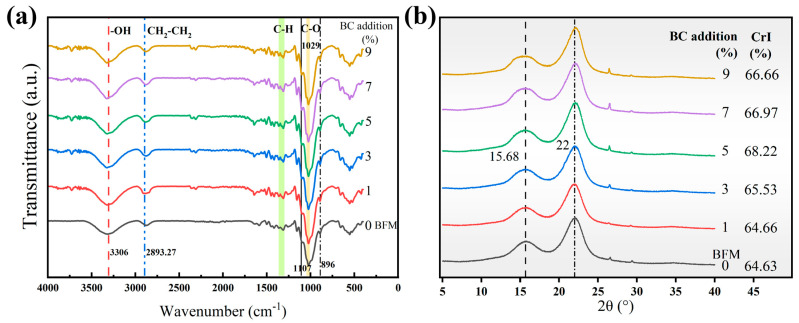
(**a**) FTIR spectrum of BFM (0%) and five groups of BC-BFMs; (**b**) XRD spectrum of BFM (0%) and five groups of BC-BFMs.

As illustrated in Figure 5a, the FTIR spectra of the six sample groups displayed a high degree of similarity, suggesting that the addition of BC did not alter the principal chemical components of BFM. The presented spectra showed two wave number regions of 3660–2800 cm^−1^ and 1750–600 cm^−1^ for observed absorption bands. The latter contained typical absorption bands attributed to the cellulose structure [50]. The peak at 3306 cm^−1^ corresponded to the stretching of the hydroxyl group [51]. The peak at 2893.27 cm^−1^ corresponded to the stretching vibrations of CH_2_–CH_2_; in the wavelength range of 1200–1500 cm^−1^, there are moderate-intensity absorptions caused by the deformation vibrations of CH, OH and CH_2_ groups. The intensity of this band was associated with the crystallinity of cellulose. The band at 896 cm^−1^ is attributed to C–C–H bending vibration, while the band at 1107 cm^−1^ corresponds to C–C stretching vibration [50]. Additionally, the absorption band at 1029 cm^−1^ was assigned to the stretching vibration of the C–O bond, which is a characteristic feature of cellulose. Notably, the intensification of the O–H stretching vibration following the addition of BC indicated enhanced interaction between the hydroxyl groups of BC and the free hydroxyl groups of cellulose, hemicellulose and lignin on the surface of bamboo fibers, facilitating the formation hydrogen bonds, leading to inter-polymer repolymerization and thereby improving inter-fiber bonding strength [33]. Although C–O bonds along the cellulose molecular chains can also participate in hydrogen bonding, the presence of hemicellulose and lignin disrupts the hydrogen bond network, resulting in a disordered, weak, and incomplete structure. The observed enhancement in C–O stretching vibration intensity upon BC addition suggested recombination between the C–O groups of BC and those of the native cellulose, contributing to enhanced hydrogen bond stability, which in turn increased vibrational resonance intensity [52].

Figure 5b illustrates the XRD spectra of the BFM and five groups of BC-BFMs. The diffraction patterns displayed three predominant characteristic peaks. The revealed peaks at 2θ = 22° and 15.68° are assigned to the (002) and (101) planes of cellulose I, respectively. [33]. As illustrated in Figure 5b, the positions and intensities of these characteristic peaks remain largely unchanged, indicating that the addition of BC does not significantly alter the inherent fiber structure of the paper matrix [44,53].

With BC addition from 1% to 5%, crystallinity increased slightly due to the formation of hydrogen bonds between BC nanofibers and bamboo fibers, especially the repolymerization of the cellulose of the nanofiber in BC itself and polymerization of cellulose between BC fiber and bamboo fiber, which resulted in the highest surface crystallinity observed in the 5% BC-BFM. When the BC addition exceeded this range, BC aggregation tended to occur. Excessive accumulation of BC may compromise interfacial bonding between the bamboo fibers. Consequently, the crystallinity decreases [54].

### 3.7. SEM Analysis

**Figure 6:**
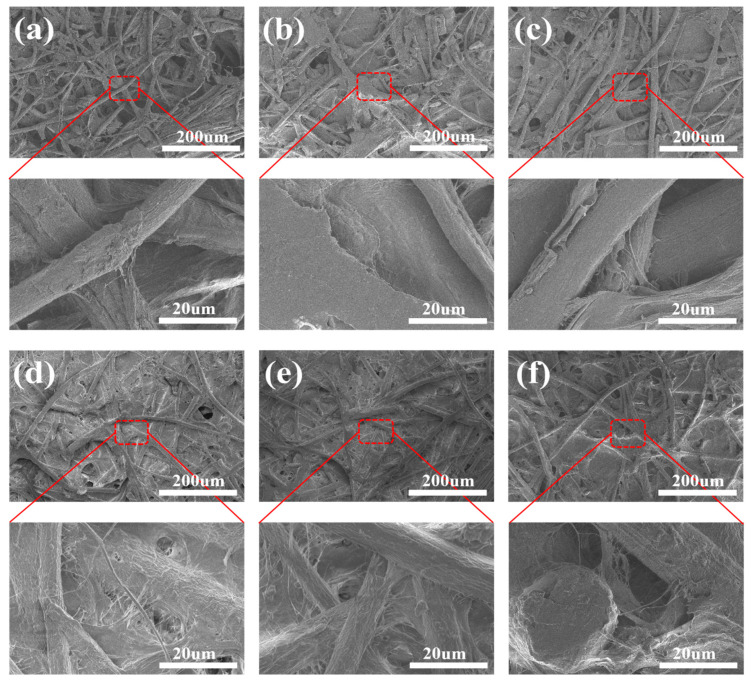
(**a**) A 0% surface micromorphology of BFM; (**b**) 1% BC-BFM; (**c**) 3% BC-BFM; (**d**) 5% BC-BFM; (**e**) 7% BC-BFM; (**f**) 9% BC-BFM.

As illustrated in Figure 6a, the surface of the BFM exhibited a rough and loosely structured morphology, primarily consisting of large bamboo fibers which were interwoven with one another. Numerous distinct pores of varying sizes and overlapping voids are observed between the fibers. This porous and irregular architecture accounts for the relatively low hydrophobicity (water contact angle: 79.73°) and high surface free energy (32.2 mJ·m^−2^) of the BFM. Figure 6b,c display the microscopic morphologies of BC-BFM incorporating 1% and 3% BC, respectively. With the addition of BC, the surface of BC-BFM becomes smoother, as the BC bridges and fills some of the smaller pores. However, the coverage remained incomplete, with many bamboo fibers still being exposed and exhibiting pronounced surface textures [55]. As shown in Figure 6d, when the BC addition reached 5%, the surface became more uniform, dense and smooth. The BC formed a complete, continuous, and mechanically robust nanofibrillar network, which constitutes the predominant surface structure [54,56]. This configuration resulted in the highest water contact angle (83.62°) and the lowest surface free energy (25.6 mJ·m^−2^), thereby achieving optimal hydrophobic performance. As can be seen in Figure 6e,f, when the BC addition was increased to 7% and 9%, the surface remained dense; however, a little surface non-uniformity began to emerge, with slight agglomeration or accumulation of BC-wrapped bamboo fibers.

**Figure 7:**
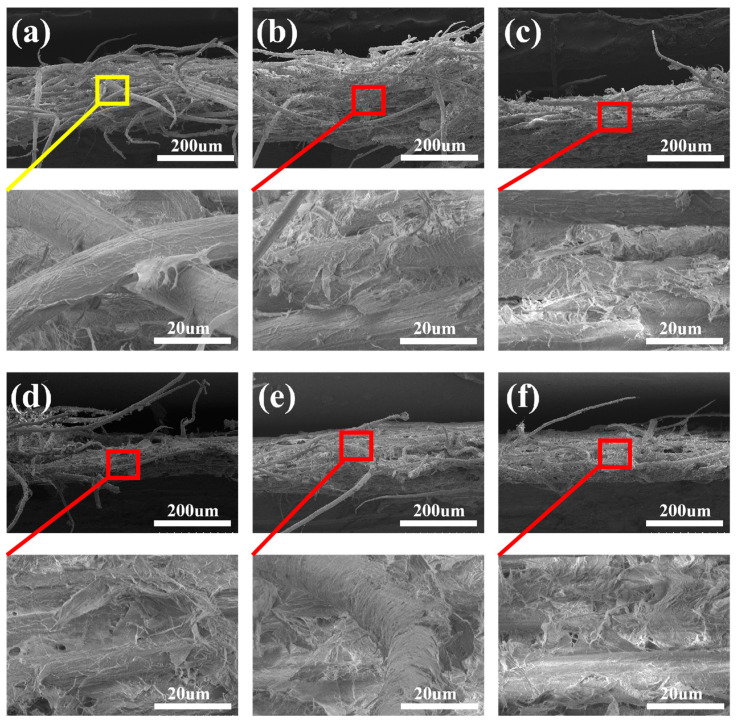
(**a**) A 0% cross-sectional micromorphology of BFM; (**b**) 1% BC-BFM; (**c**) 3% BC-BFM; (**d**) 5% BC-BFM; (**e**) 7% BC-BFM; (**f**) 9% BC-BFM.

Figure 7a illustrates the presence of extensive fiber pull-out from the BC-BFM, with an irregular fracture surface exhibiting typical characteristics of brittle fracture, consistent with the material’s relatively low mechanical strength. Figure 7b,f demonstrate that upon the addition of BC at concentrations ranging from 1% to 9%, the fracture surfaces gradually transition to a smoother and denser morphology. The nanofibers of BC formed a continuous network between the bamboo fibers, effectively wrapping bamboo fiber and bridging microcracks and filling interstitial pores, which was correlated directly with the significant enhancement in mechanical strength [57].

### 3.8. Germination Experiment of BC-BFM

**Figure 8:**
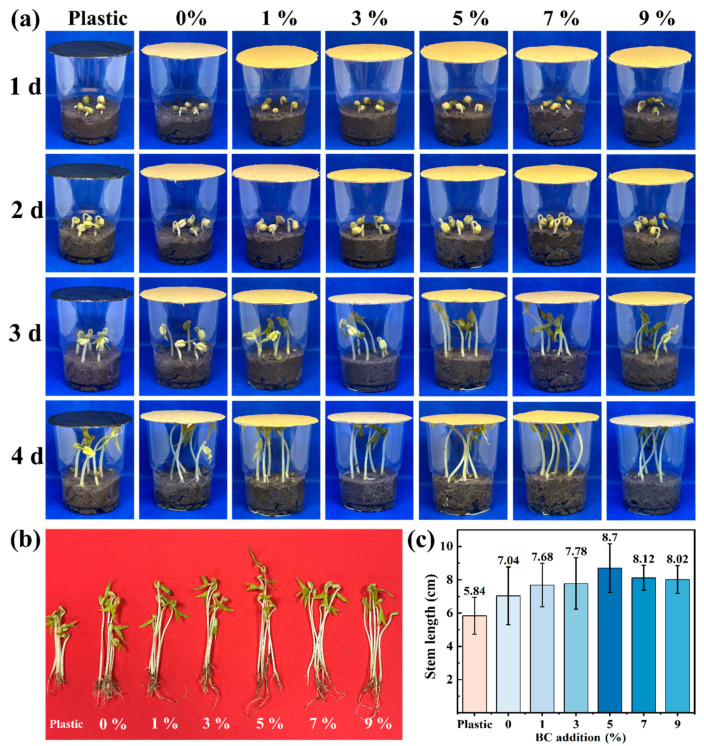
(**a**) Growing image of mung bean cultivation with plastic mulch and BC-BFM coverings. (**b**) Harvested mung bean sprouts. (**c**) Stem length of mung bean sprouts after 4 d of growth.

Figure 8 illustrates the seed germination test procedure and stem elongation of mung seedlings using plastic, BFM and five groups of BC-BFMs over a 4 d period. As shown in Figure 8a, mung seedlings under BFM and BC-BFM exhibited faster growth than those under the plastic film. This should be primarily attributed to the relatively lower air permeability of the black plastic film, which restricted quick air exchange [43]. As demonstrated in Figure 8b, at a 5% BC addition, the average sprout stem height reached a maximum of 8.7 cm. This may be because the oxygen and moisture content in sealed space and the air exchanging speed with the surrounding environment were affected by BC-BFM with different BC additions, which further affected the growth speed of sprouts.

### 3.9. Degradability of BC-BFM

**Figure 9:**
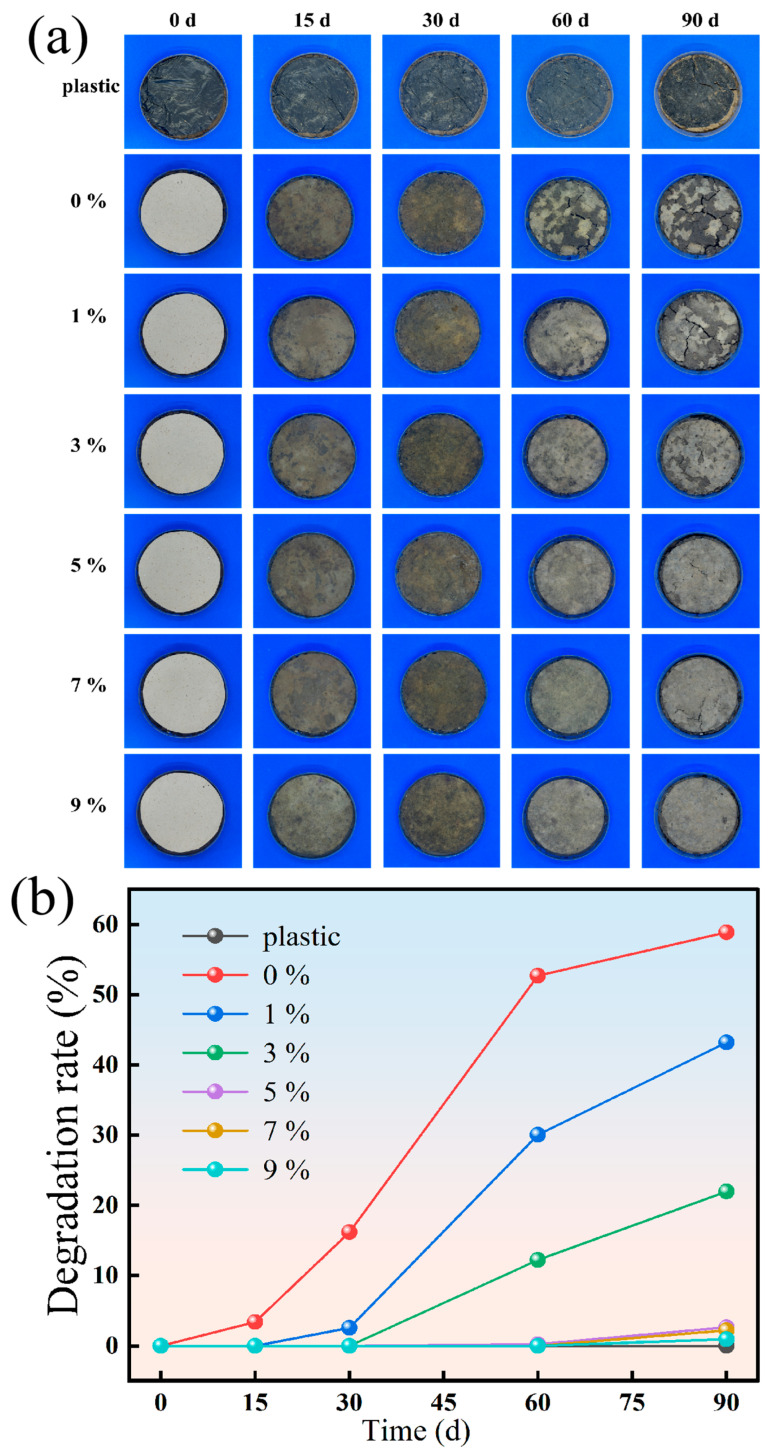
(**a**) Degradable morphology. (**b**) Degradation rate of plastic. BFM and five groups of BC-BFMs.

The degradable morphology of plastic, BFM and five groups of BC-BFMs is shown in Figure 9a. The degradation rate of plastic, BFM and five groups of BC-BFMs is shown in Figure 9b.

As shown in Figure 9a, plastic mulch exhibited no surface degradation over the 90 d test period due to its chemically stable structure; only minor cracking and color fading were observed, confirming it as non-degradable mulch. In contrast, BFM without BC addition began to degrade significantly after 30 d, with hemicellulose being the most microbially susceptible component being preferentially broken down. As shown in Figure 9b, by 90 d, the degradation rate of BFM neared 60%. With increasing BC addition, a robust hydrogen-bonding network forms between BC and bamboo fibers. This interaction not only enhances the mechanical strength of the composite but also hinders soil microorganisms from accessing and disrupting the fiber junctions, thereby slowing the degradation process. Consequently, the degradation area of BC-BFM progressively decreased by 90 d as the BC addition increased. BC-BFM with 1% and 3% BC addition indicated that it was still degradable in essence; although the degradation rate slowed down, the microbial community in the soil can eventually gradually decompose BC-BFM.

**Figure 10:**
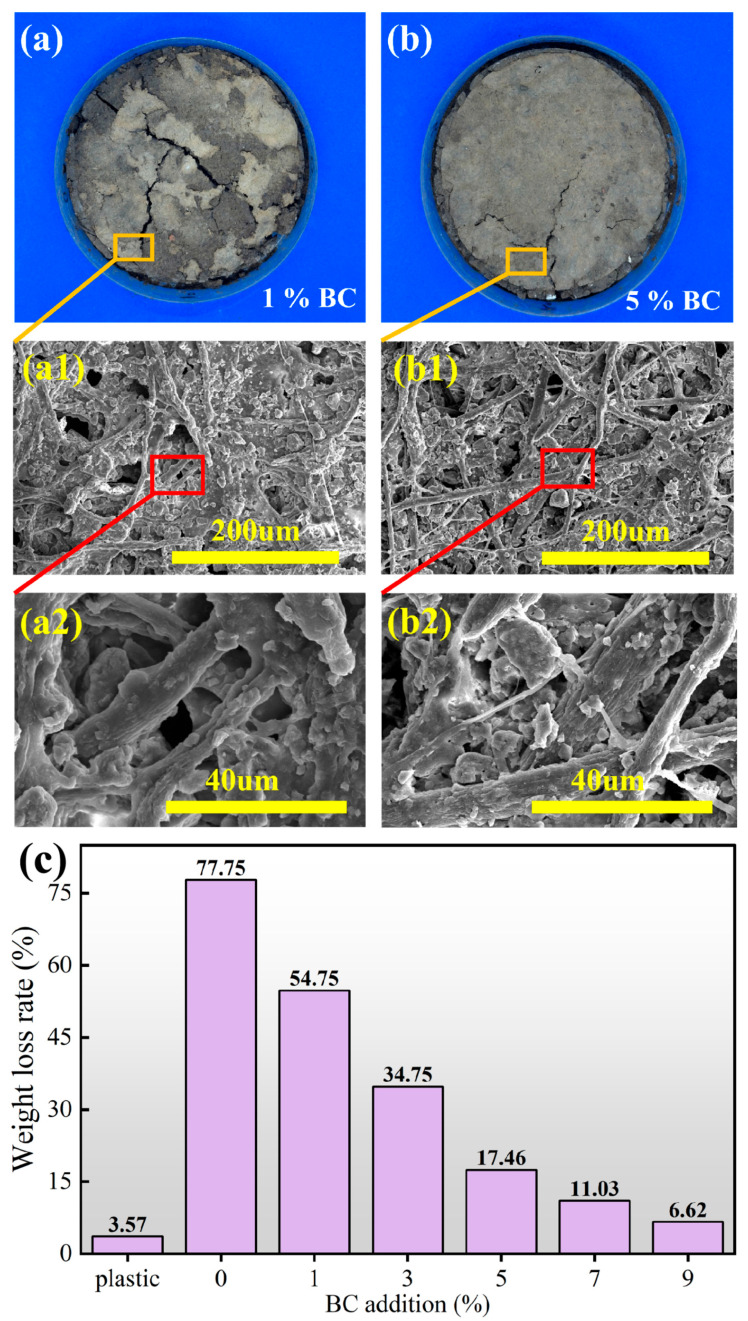
(**a**) BC-BFM with 1% BC after 90 d of degradation. (**b**) BC-BFM with 5% BC after 90 d of degradation. (**c**) WLR after 90 d of degradation.

Figure 10a shows that the BC-BFM group with 1% BC addition exhibited the fastest degradation. In Figure 10(a1), the degraded sample surface displays uneven erosion characteristics with visible cracks and holes between the fibers. Figure 10(a2) reveals severe damage to the fiber structure, which had disintegrated into small fragments, indicating that microorganisms in soil preferentially attacked the less stable interfacial regions between bamboo fibers and BC fiber. Figure 10b presents images after 90 d of degradation for the sample with 5% BC addition. Macroscopically, no obvious degradation was observed. As shown in Figure 10(b1), BC fibers completely filled the gaps between bamboo fibers, forming a continuous network that effectively slowed microbial invasion. Although some microscale pores appeared between fibers, indicating that invisible biodegradation occurred slowly and steadily, the degradation rate of the 5% BC group was slower than that of the 1% BC group.

The weight loss rate of the BC-BFM and plastic film after 90 d is shown in Figure 10c. The degradation weight loss rate of all BC-BFMs was significantly higher than that of plastic films (the weight loss rate of the plastic group was only 3.57%). The 0% BC addition showed the highest weight loss rate (77.75%), indicating that it was the most easily decomposed by microorganisms in soil. With the increase in the addition of BC, the weight loss rate of BC-BFM showed a continuous downward trend: from 54.75% at 1% BC to 6.62% at 9% BC. This result was consistent with the trend observed in Figure 9b. Notably, the BC-BFM with 9% BC demonstrated the lowest weight loss rate, which nearly twice that of plastic film after 90 d of degradation.

Therefore, the addition of BC effectively ensured that the mulch maintained functional integrity throughout the longer growth cycle. This extended its service life to more than three times than that of BFM, transforming it into a high-performance, degradable mulch with adjustable lifespan by adjusting the addition of BC and enhancing its agronomic utility.

## 4. Conclusions

High-performance biodegradable bamboo fiber mulch films were fabricated by incorporating bacterial cellulose (BC) into bamboo fiber pulp, which was pretreated using a combined bio-fermentation and low-alkali process. BC was added at varying additions ranging from 1% to 9%. The incorporation of BC significantly enhanced the mechanical properties of the resulting BC-BFM films. Compared to the control, the film with 9% BC exhibited a 51.09% increase in tensile index, a 31.76% increase in burst index, and a 28.49% increase in tear index. SEM observations revealed that BC nanofibers were effectively distributed within the gaps between bamboo fibers, filling both macroscale and microscale voids and forming an intertwined network. FTIR and XRD analyses further demonstrated that the addition of BC established a dense hydrogen bonding network, promoting repolymerization of cellulose with the bamboo fibers, which contributed to an increase in the crystallinity of the composite films.

In a mung bean seed germination test, the BC-BFM with 5% BC addition promoted significantly greater sprout height (8.70 cm) compared to conventional plastic mulch (5.84 cm), representing a 48.9% increase. The 90 d soil degradation test confirmed the biodegradability of all BC-BFM formulations. The degradation rate of BC-BFM within 90 d was three times less than that of BFM. The weight loss rate decreased progressively with higher BC content, from 54.75% at 1% BC to 6.62% at 9% BC—all remaining higher than that of the plastic film. Notably, the service life of BC-BFM was extended to more than three times that of BFM without BC.

These results demonstrate that BC serves as an effective nano-reinforcement agent, enhancing the mechanical properties, hydrophobicity, and plant-growth-promoting capacity of BC-BFM by optimizing its micro–nano structure.

## Data Availability

The raw data supporting the conclusions of this article will be made available by the authors on request.
